# Whey-Adapted versus Natural Cow’s Milk Formulation: Distinctive Feeding Responses and Post-Ingestive c-Fos Expression in Laboratory Mice

**DOI:** 10.3390/foods11020141

**Published:** 2022-01-06

**Authors:** Erin L. Wood, Sarah N. Gartner, Anica Klockars, Laura K. McColl, David G. Christian, Robin E. Jervis, Colin G. Prosser, Elizabeth A. Carpenter, Pawel K. Olszewski

**Affiliations:** 1 Faculty of Science and Engineering, University of Waikato, Hamilton 3240, New Zealand; erin.lavinia.wood@icloud.com (E.L.W.); sarahgartner1@gmail.com (S.N.G.); anica.klockars@waikato.ac.nz (A.K.); Laura.mccoll@waikato.ac.nz (L.K.M.); dchristian@tutanote.com (D.G.C.); robinj1997@hotmail.com (R.E.J.); 2 Dairy Goat Cooperative, Ltd., Hamilton 3240, New Zealand; Colin.prosser@dgc.co.nz (C.G.P.); Liz.Carpenter@dgc.co.nz (E.A.C.); 3 Department of Food Science and Nutrition, University of Minnesota, Saint Paul, MN 55113, USA; 4 Department of Integrative Biology and Physiology, Medical School, University of Minnesota, Minneapolis, MN 55455, USA

**Keywords:** brain, satiety, feeding, palatability, milk, formula, animal models

## Abstract

The natural 20:80 whey:casein ratio in cow’s milk (CM) for adults and infants is adjusted to reflect the 60:40 ratio of human milk, but the feeding and metabolic consequences of this adjustment have been understudied. In adult human subjects, the 60:40 CM differently affects glucose metabolism and hormone release than the 20:80 CM. In laboratory animals, whey-adapted goat’s milk is consumed in larger quantities. It is unknown whether whey enhancement of CM would have similar consequences on appetite and whether it would affect feeding-relevant brain regulatory mechanisms. In this set of studies utilizing laboratory mice, we found that the 60:40 CM was consumed more avidly than the 20:80 control formulation by animals motivated to eat by energy deprivation and by palatability (in the absence of hunger) and that this hyperphagia stemmed from prolongation of the meal. Furthermore, in two-bottle choice paradigms, whey-adapted CM was preferred against the natural 20:80 milk. The intake of the whey-adapted CM induced neuronal activation (assessed through analysis of c-Fos expression in neurons) in brain sites promoting satiation, but importantly, this activation was less pronounced than after ingestion of the natural 20:80 whey:casein CM. Activation of hypothalamic neurons synthesizing anorexigenic neuropeptide oxytocin (OT) was also less robust after the 60:40 CM intake than after the 20:80 CM. Pharmacological blockade of the OT receptor in mice led to an increase in the consumption only of the 20:80 CM, thus, of the milk that induced greater activation of OT neurons. We conclude that the whey-adapted CM is overconsumed compared to the natural 20:80 CM and that this overconsumption is associated with weakened responsiveness of central networks involved in satiety signalling, including OT.

## 1. Introduction

Nutritional benefits of cow’s milk (CM) consumption, one of the most common elements of daily diets on a global scale, are to a large extent associated with its protein composition [1,2,3,4]. Whey and casein are the predominant components of the protein fraction [5]. However, the protein fraction of CM and other kinds of animal milk (e.g., goat’s milk (GM)) contains the natural whey:casein ratio of about 20:80, which is in contrast to the 60:40 ratio of human milk [6]. Many milk products for human nutrition—most commonly infant formulas—have therefore whey added in order to parallel the 60:40 ratio of human milk [7,8,9].

While milk proteins, in general, affect metabolic parameters [1,5,10], whey and casein each independently produce distinct responses by interacting with gut transporters and receptors, modifying gastric emptying, and changing gut endocrine activity [1,5,11,12]. Casein is digested more slowly than whey [13,14], which delays metabolite delivery to the intestine [11,15,16,17] and consequently leads to fast and pronounced amino acid level increases after whey intake [11,18,19] and delayed and prolonged hyperaminoacidemia after casein [11]. This translates to differences in hormonal release, having downstream effects on the brain. For example, both casein and whey promote hypophagia via PPY [20,21], but whey stimulates the release of CCK, GLP-1 and GIP more robustly [16,19,22,23]. Whey proteins affect serotoninergic activity [24,25]. Finally, ingestion of either whey or casein generates changes in feeding [16,19,26,27].

The aforementioned reports focus on the effects of whey or casein alone. One should not apply an oversimplified assumption that the combined whey and casein, while being part of the complex milk-based diet, produce appetite and metabolic effects that are either negligible or merely ‘proportional’ to the whey:casein content. Also, the animal source of a given milk formulation is extremely likely to be a modifying factor as milk derived from different species possess unique characteristics [28].

Yet despite this frequent modification to the protein fraction, surprisingly little is known about how the shift from the natural to the whey-enhanced ratio in commonly consumed kinds of milk (importantly, in CM), affects appetite and post-ingestive processes. El Khoury et al., who gave adults a 20:80 or whey-adapted 60:40 milk beverage, found that the whey-adapted milk consumed with cereal reduced postprandial glycemia independently from insulin, mainly via delayed gastric emptying [22]. The preprandial glucose was lower and GLP-1 release was elevated after the 60:40 milk. In animal studies, obese rats displayed better glucose tolerance when eating whey instead of whey plus casein [29,30].

In our recent mouse study, which was the first to directly examine how the 20:80 versus 60:40 whey:casein milk intake affects feeding and feeding-related neural mechanisms, the whey-adapted formulation was found to be overconsumed, and this avid consumption was associated with differences in brain activation in regions controlling appetite and in melanocortin mRNA levels [31]. Importantly, this initial study was carried out using goat’s milk, whose effects on numerous physiological parameters, from gastrointestinal to sensory to immune, is different from cow’s milk. Thus, though the above-mentioned murine study was informative in determining that whey-adapted GM produces different appetite-related effects from the natural control (20:80) formulation, it is unknown whether the same whey:casein ratio enhancement in CM, the most commonly used milk worldwide, would have similar consequences for appetite.

Thus, the current study, designed to bridge this gap, investigated whether the whey-adapted (60:40) CM formulation was consumed in different quantities than the control natural 20:80 CM by laboratory mice. In the single tastant (no diet choice) scenarios, we studied natural control vs. whey-adapted CM intake in animals motivated to eat mainly by calorie needs (after energy deprivation) or by palatability (in non-deprived individuals). In two-bottle choice tests, relative preference for the 60:40 vs. natural control CM formulation was assessed.

Taking into account the earlier report showing that the 60:40 GM is preferred over the 20:80 GM and that the relative preference for GM is higher than for CM, we examined whether whey-adapted CM is not only preferred over the natural control CM, but also over the natural 20:80 GM formulation. We hypothesized that appetite responses to the 20:80 vs. 60:40 CM stem from the central mechanisms being distinctively affected by whey:casein ratio enhancement. Therefore, activation of feeding-related brain circuitry (determined through analysis of c-Fos expression) was analysed in animals just after the completion of a 1-h meal in which similar amounts of the control 20:80 vs. 60:40 CM were ingested. 

In order to further substantiate the hypothesis that greater consumption of the 60:40 CM stems—at least to some extent—from suppressed satiety signalling in the brain, we examined whether the percentage of c-Fos-positive (activated) neurons synthesizing a key satiety neuropeptide, oxytocin (OT) is lower after intake of the 60:40 than 20:80 whey:casein CM. Typically, the level of activation of hypothalamic OT neurons parallels the magnitude of a satiety response [32]. We subsequently tested whether pharmacological blockade of the receptor that binds OT elevates consumption of the 20:80 CM but not 60:40 CM, thus of the formulation which activates the anorexigenic OT system in a more robust fashion. Pharmacological blockade of the OT receptors was done with the antagonist that penetrates the blood-brain barrier, L-368,899 [33,34].

## 2. Materials and Methods

### 2.1. Animals

The studies were performed on adult male C57Bl mice. The species/strain/age combination was chosen as one of the most frequently used food intake laboratory animal models that had been studied also in relation to goat milk formulations differing in whey:casein ratios (feeding behaviour and gene expression), OT receptor blockade and diet preference, and c-Fos and feeding [33,34,35]; thus, it can be treated as a reliable reference model for the data obtained here. The animals were housed individually in a temperature- and humidity-controlled (22 °C; relative humidity 40–45%) facility with a 12:12 h light:dark schedule (lights on at 09:00). The mice had unlimited access to standard laboratory chow (Diet 86, Sharpes Stock Feed, Wairarapa, New Zealand) and water unless stated otherwise. Chow was presented in the overhead hopper, whereas water, in a 150-millilitre bottle with a 10-cm nozzle, was placed in the conventional overhead lid bottle holder. Milk formulations (DGC, Ltd., Hamilton, New Zealand) were available periodically as per the detailed description below. A different cohort of animals was used in each study unless noted otherwise. The procedures had received prior approval by the University of Waikato’s institutional animal ethics committee (approval #1057).

### 2.2. Milk Formulations Used in the Studies

The control CM formula contained the natural whey:casein protein ratio of 20:80 (Control 20:80), whereas the whey-adapted CM test formula had 60% whey and 40% casein (60:40). The composition of the prepared solutions can be found in Table 1. In addition to CM formulations, in Experiment Section 2.3.4, we used equivalent whey:casein ratio GM-based formulations that had been previously tested in laboratory animal feeding trials [31]. All formula compositions follow Codex standard (CODEX STAN 72-1981; based on nutrients/energy value), which requires lactose to be within the range of 5.4–9.8 g/100 mL. The solutions were prepared just before use by being reconstituted in tap water. In order to avoid neophobia, all mice were pre-exposed to the milk on at least two occasions (1 h each) within two weeks prior to the trials. Milk was given to the animals in 150-millilitre bottles equipped with 10-cm-long metal nozzles. The bottles were placed manually at the scheduled time of fluid presentation in the standard cage lid bottle holder at a 45-degree angle. The positioning allowed animals to have unobstructed access to the nozzles.

### 2.3. Feeding Studies

#### 2.3.1. Energy Deprivation-Induced Intake of Whey-Adapted vs. Control CM Formulation

Before gaining episodic access to a single bottle of milk, mice (*n* = 10/group; individually housed) were deprived overnight of standard chow (water was available during the time of energy deprivation). At 09:00 a bottle containing either the Control 20:80 whey:casein CM formulation or the whey-adapted 60:40 CM was placed in the cage for 3 h. Water was removed for the 3-h meal. Milk consumption was measured in grams at 1 and 3 h.

#### 2.3.2. Intake of Whey-Adapted vs. Control CM Formulation in Non-Deprived Animals

The same cohort of animals as in Section 2.3.1 was used here (7 days of no treatment elapsed between studies). On the day of the study, standard chow and water were removed from cages (at 09:00) and a bottle containing either the Control 20:80 whey:casein CM formulation or the whey-adapted 60:40 CM (*n* = 10/group; individually housed) was placed in the cage for 3 h. The formulations were the only source of calories and fluid for the 3-h meal. The amount of consumed formulations (in grams) was assessed at 1 and 3 h.

#### 2.3.3. Preference for the Simultaneously Presented Whey-Adapted vs. Control CM Formulation (Two-Bottle Choice)

Mice (*n* = 7–8/group; individually housed) were acclimatized to the two-bottle presentation of the Control 20:80 whey:casein CM formulation and the whey-adapted 60:40 CM on two separate occasions one week prior to the study. On the experimental day at 10:00, standard chow and water were removed and animals were simultaneously presented with two bottles, one containing the Control 20:80 CM formulation and the other, the 60:40 CM. Intake was measured after 2 h by weighing the bottles and the data were expressed in grams.

#### 2.3.4. Preference for the Simultaneously Presented Whey-Adapted vs. Control CM and GM Formulations (Two-Bottle Choice)

In earlier laboratory animal studies [35], we have found that GM is more preferred to CM. We have also reported that whey-adapted GM is consumed more avidly than the natural 20:80 GM formulation [31].

Here, by using two-bottle tests (the same as described above in Section 2.3.3 2 h, water and chow removed during the test) in separate cohorts of mice (*n* = 7/group; individually housed), we investigated whether the whey-adapted formulations are preferred over the natural (20:80) whey:casein milks regardless of which species the milk was derived from. In other words, we evaluated whether the enhanced whey:casein ratio in CM supersedes the gustatory effect of milk derived from the previously evaluated GM.

We first confirmed that the whey-adapted GM is indeed more preferred than the Control 20:80 GM. Then the two-bottle choice test was done in mice that received access to the control vs. whey-adapted milk on the experimental day (Control 20:80 CM vs. whey-adapted 60:40 GM; Control 20:80 GM vs. whey-adapted 60:40 CM). Finally, we examined preference when formulations containing the same whey:casein ratio were presented simultaneously (Control 20:80 CM vs. Control 20:80 GM; whey-adapted 60:40 CM vs. whey-adapted 60:40 GM). Formulation intake after 2 h was measured in grams.

### 2.4. c-Fos Expression in the Feeding-Related Brain Circuit after Consumption of the Same Amount of the Control 20:80 versus Whey-Adapted CM Formulation

As whey-adapted CM promoted more avid consumption (elevated preference compared to the natural 20:80 control and in the no-choice scenarios, a prolonged meal) we wished to examine whether consumption of the same amount of the Control 20:80 CM vs. the whey-adapted 60:40 CM formulation led to a different level of neuronal activation of brain areas that control eating behaviour. For the analysis, we chose regions that participate in the regulation of feeding for energy (hunger, satiation) and pleasure [36]: the medial preoptic area, paraventricular nucleus, supraoptic nucleus, arcuate nucleus, ventromedial hypothalamic nucleus, dorsomedial hypothalamic nucleus, lateral hypothalamic area, dorsal motor nucleus of the vagus, nucleus of the solitary tract, area postrema, bed nucleus of the stria terminalis, nucleus accumbens shell, nucleus accumbens core, and central nucleus of the amygdala. A different pattern of neuronal activation after consumption of a similar amount of milk would shed more light on whether the readiness of animals to consume more food given the whey-adapted formulation stems from dysregulation of hunger/satiety signalling and/or different reward processing.

Neuronal activation was determined by immunohistochemical detection of an immediate-early gene product, c-Fos. Maximum c-Fos immunoreactivity (IR) occurs about 60–90 min after the stimulus (see, e.g., [37,38]). In our study, we examined c-Fos IR corresponding to the beginning of the meal (to establish baseline activity without food consumption) and after 1-h exposure to the Control 20:80 vs. whey-adapted 60:40 CM.

On the experimental day, chow and water were removed from the cages at 10:00. The animals (*n* = 7–8/group) were divided into three groups: Group 1 did not receive any milk and was perfused an hour later in order to visualize baseline c-Fos IR corresponding to the beginning of the meal. Group 2 was given a bottle containing the Control 20:80 CM for 1 h. Group 3 received the whey-adapted 60:40 CM formulation for 1 h. Bottles were removed after 1 h, but no food or water was returned to the cages until the animals from Groups 2 and 3 were perfused an hour later (i.e., at 12:00). Since the intake of the Control vs. whey-adapted CM formulations does not differ during the first hour of consumption, but instead, the intake of the whey-adapted CM is prolonged, the mice from both groups consumed similar amounts of the milk during the hour (2.4 ± 0.15 g).

Mice were anaesthetized (urethane, 35% i.p.) and perfused with room-temperature saline (10 mL) followed by ice-cold 4% paraformaldehyde (PFA; 50 mL) in 0.1 M phosphate buffer (pH 7.4). Brains were excised and postfixation was done overnight in PFA (4 °C). Coronal 60 μm vibratome (Leica, Dusseldorf, Germany) free-floating sections were processed immunohistochemically for c-Fos. The sections were first incubated in 3% H_2_O_2_ in 10% methanol in TBS (10 min), then for 18 h in the rabbit anti-c-Fos antibody (1:3000; Synaptic Systems, Göttingen, Germany; 4 °C). The tissue was incubated subsequently for 1 h in the secondary biotinylated antibody (goat-anti-rabbit 1:400; Vector Laboratories, Burlingame, CA, USA) followed by a 1-h incubation in the avidin-biotin complex (1:800; Vector Laboratories, USA) at room temperature. 0.05% DAB, 0.01% H_2_O_2_ and 0.2% nickel sulphate (Sigma, Saint Louis, MO, USA) were used in the final step to visualize staining. All incubations were performed in 0.25% gelatin and 0.5% Triton X-100 (Sigma, Saint Louis, MO, USA) in TBS. Sections were mounted onto slides, air-dried, dehydrated in ethanol, soaked in xylene, and embedded in Entellan (Merck, Darmstadt, Germany). c-Fos IR nuclei were counted bilaterally in the regions (3–4 sections/animal). Densities of c-Fos positive nuclei per mm^2^ of the tissue were averaged per group.

### 2.5. Activation of Oxytocin (OT) Neurons after Consumption of the Same Amount of the Control 20:80 versus Whey-Adapted CM Formulation

Having noted differences in c-Fos immunoreactivity in the hypothalamus, we examined whether the percentage of activated (i.e., c-Fos-positive) neurons synthesizing an anorexigen, oxytocin (OT), differs after consumption of the same amount of the Control 20:80 versus whey-adapted milk. This was done in order to assess whether OT, one of the key neuropeptidergic satiety systems, is engaged more after consumption of one diet versus the other.

In order to visualize c-Fos expressing OT cells, after the completion of the c-Fos staining, hypothalamic sections containing the paraventricular (PVN) and supraoptic (SON) nuclei (thus, the two regions where OT cells are amassed [32]) were further stained for visualization of OT. The immunohistochemical procedure was similar to the one described above. For the primary antibody incubation, rabbit anti-OT was used (1:25,000; Millipore, Temecula, CA, USA), and nickel sulphate was not added to the DAB reagent. Consequently, OT neurons were stained brown instead of the black colour obtained for c-Fos.

In the c-Fos+OT staining, we determined the total number of OT neurons (this total number of OT neurons is typically unaffected by food intake) and the number of OT neurons positive for c-Fos (in earlier studies, this has been found to be very low at the beginning of a meal and it gets elevated as the amount of ingested food increases [39]). Cells were counted bilaterally in the PVN and SON, and the percentage of OT neurons containing Fos-positive nuclei was calculated.

### 2.6. Effect of Pharmacological Blockade of the OT Receptor on Consumption of Whey-Adapted vs. Control CM Formulation in Non-Deprived Animals

It has been previously reported that peripheral administration of the blood-brain barrier-penetrant OT receptor antagonist, L-368,899, in nondeprived mice elevates consumption of diets whose intake produces a particularly robust activation of the hypothalamic OT system [34]. Thus, considering that the Control 20:80 formulation generated a greater percentage of c-Fos-positive OT neurons in the PVN (in the c-Fos+OT experiment above), we sought to investigate whether consumption of this milk is differently affected by L-368,899 than intake of the whey-adapted formulation.

On the day of the study, chow and water were removed from cages (at 09:00) and a bottle containing either the Control 20:80 whey:casein CM formulation or the whey-adapted 60:40 CM was placed in the cage for 3 h. The formulations were the only source of calories and fluid for the 3-h meal. Ten minutes prior to the presentation of either diet, the mice were injected intraperitoneally (IP) with isotonic saline (vehicle) or 1 mg/kg b. wt. L-368,899 (the dose known to elevate intake of diets that induce the most robust response of the central OT system [33,34]). The amount of consumed formulations (in grams) was assessed at 3 h for saline vs. L-368,899-treated groups (*n* = 9–11 per group) separately in the cohort given the Control 20:80 CM and in the cohort given the whey-adapted 60:40 milk.

### 2.7. Statistical Analyses

Feeding was standardized per gram of body weight. Food intake and immunohistochemistry two-group comparisons were analysed with Student’s *t*-test. In the feeding studies where three groups were compared with each other, a one-way ANOVA followed by Tukey’s post-hoc test with a correction for multiple comparisons was used. Differences were considered significant for *p* < 0.05.

## 3. Results

**Figure 1:**
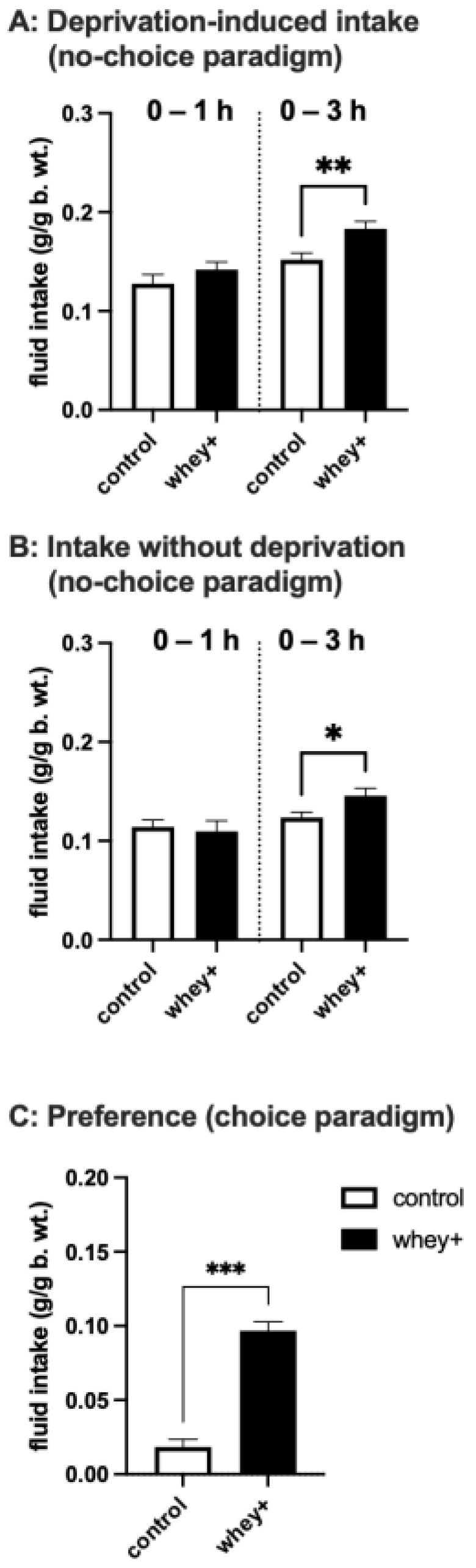
Intake of the individually presented natural Control (20:80 whey:casein) CM and the whey-adapted (60:40 whey:casein; whey+) CM in (**A**) overnight-deprived mice and (**B**) non-deprived mice during 3-h access to the diets. Intake was measured at 1 and 3 h. (**C**) Intake of simultaneously presented Control (20:80) CM and whey-adapted (60:40) CM during a 2-h two-bottle choice test. * *p* ≤ 0.05; ** *p* ≤ 0.01; *** *p* ≤ 0.001.

In the first hour of a meal in the no-choice feeding scenarios, both overnight-deprived animals and non-deprived mice drank similar amounts of the Control 20:80 and the whey-adapted 60:40 CM (Figure 1A,B). The majority of feeding activity occurred during this first hour. However, in the next 2 h of milk availability, animals given the whey-adapted CM formulation continued consumption at a level higher than those given the Control 20:80 milk. This was the case for the hungry mice (*p* = 0.007) eating primarily for energy as well as the non-deprived ones (*p* = 0.029) that were eating mainly for palatability.

**Figure 2:**
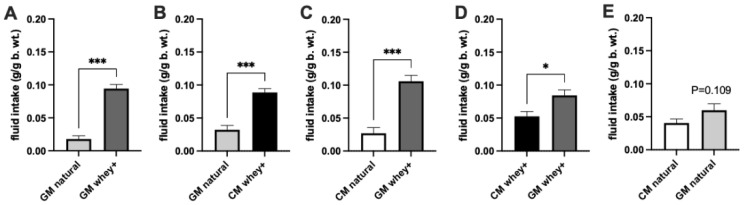
Consumption of simultaneously presented CM- and GM-based formulations with the natural control (20:80) and whey-adapted (60:40) whey:casein ratio. Non-deprived animals were given access to two bottles over a 2-h episodic exposure: (**A**) GM natural control and GM whey-adapted, (**B**) GM natural control and CM whey-adapted, (**C**) CM natural control and GM whey-adapted, (**D**) CM whey-adapted and GM whey-adapted and (**E**) CM natural control and GM natural control. * *p* ≤ 0.05; *** *p* ≤ 0.001.

In the two-bottle choice test, the animals drank approximately four times more of the whey-adapted CM than its natural 20:80 CM equivalent (*p* < 0.001; Figure 1C). This higher preference for the CM was paralleled in the preference experiment utilizing GM-based formulations: the mice chose the 60:40 whey:casein ratio (*p* < 0.001; Figure 2A). The whey-adapted CM was also preferred over the natural Control 20:80 GM (*p* < 0.001; Figure 2B), and the whey-adapted GM was chosen over the natural Control 20:80 CM (*p* < 0.001; Figure 2C). Finally, when whey:casein content-matched formulations were presented simultaneously, mice showed preference for the GM-based ones: significant for the 60:40 GM vs. 60:40 CM (*p* = 0.0105; Figure 2D) and a trend toward significance for the natural Control 20:80 GM vs. 20:80 CM (*p* = 0.109; Figure 2E).

**Figure 3:**
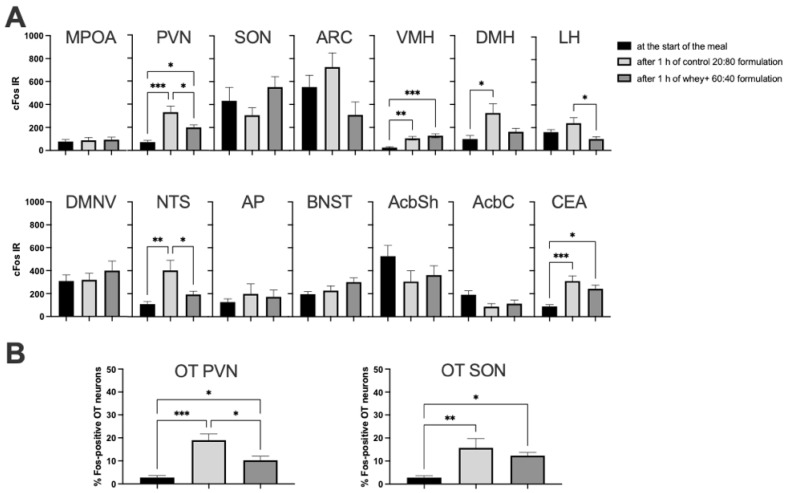
c-Fos immunoreactivity in feeding-related brain sites (**A**) and in OT neurons (**B**) corresponding to the start of the meal (baseline-black bars) or after 1 h of ingesting a similar amount of the Control 20:80 whey:casein CM (light grey bars) or of the 60:40 whey-casein (whey+) CM. MPOA—medial preoptic area; PVN—paraventricular nucleus; SON—supraoptic nucleus; ARC—arcuate nucleus; VMH—ventromedial hypothalamic nucleus; DMH—dorsomedial hypothalamic nucleus; LH—lateral hypothalamic area; DMNV-dorsal motor nucleus of the vagus; NTS—nucleus of the solitary tract; AP—area postrema; BNST—bed nucleus of the stria terminalis; AcbSh—nucleus accumbens shell; AcbC—nucleus accumbens core; CEA—central nucleus of the amygdala. * *p* ≤ 0.05; ** *p* ≤ 0.01; *** *p* ≤ 0.001.

**Figure 4:**
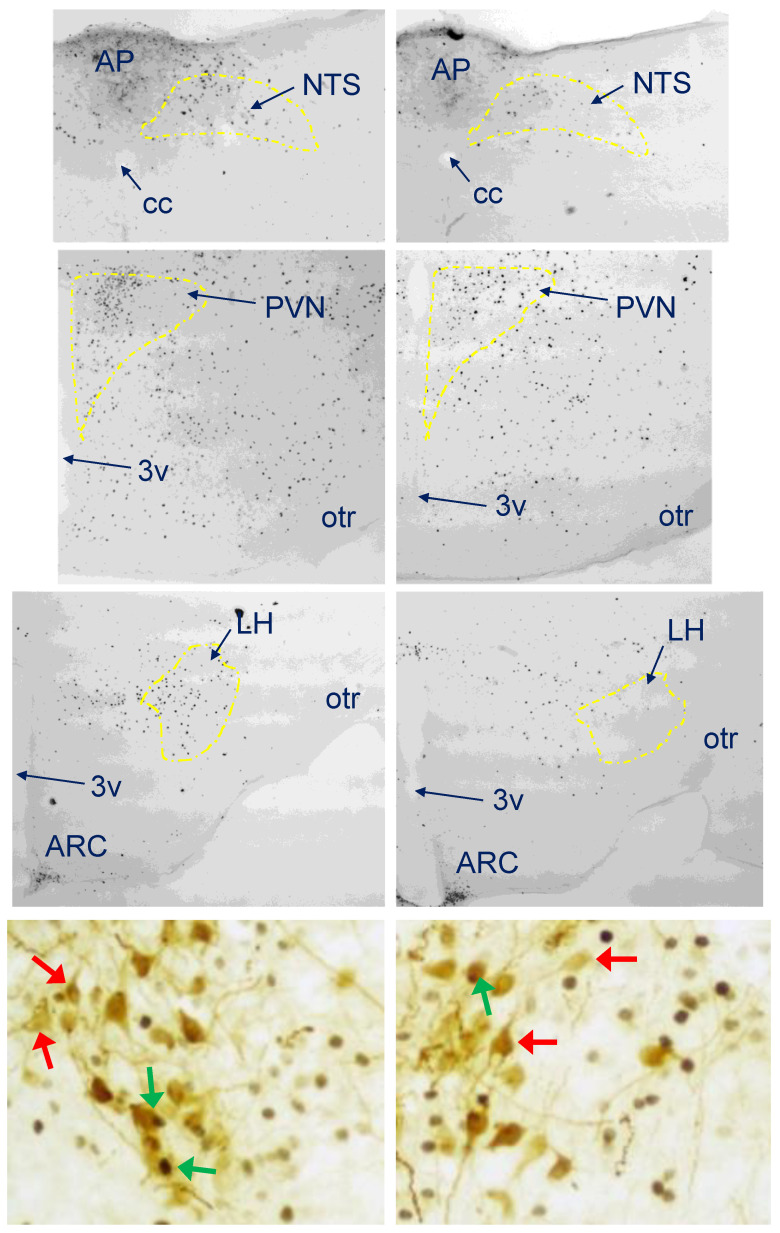
Photomicrographs depicting feeding-related brain sites (top three rows) and PVN OT neurons (bottom row) in which c-Fos immunoreactivity was different after ingestion of the same volume of the control 20:80 CM (left panel) vs. 60:40 (right panel) whey:casein milk formulation. 3v—third ventricle, AP—area postrema, ARC—arcuate nucleus, cc—central canal, NTS—nucleus of the solitary tract, otr—optic tract, LH—lateral hypothalamus; PVN—paraventricular hypothalamic nucleus; in the double-stained sections, red arrows point to OT cells devoid of c-Fos, whereas green arrows indicated c-Fos-positive OT neurons.

Compared to the baseline (at meal onset), CM formulation consumption led to changes in the activity of several brain areas related to feeding (Figure 3A). An increase in the number of c-Fos-positive nuclear profiles regardless of the whey:casein ratio was found in the hypothalamic paraventricular (PVN; for the Control, *p* = 0.001; for the whey-adjusted, *p* = 0.0468) and ventromedial nuclei (VMH; for the Control, *p* = 0.0032; for the whey-adjusted, *p* = 0.0003) and in the central nucleus of the amygdala (CEA; for the Control, *p* = 0.0006; for the whey-adjusted, *p* = 0.0143). However, in the PVN, the level of c-Fos immunoreactivity was significantly higher (two-fold) in the group consuming the Control 20:80 than the whey-adapted diet (*p* = 0.0312). In the dorsomedial hypothalamic nucleus (DMH), only the intake of the natural Control 20:80 CM led to a significant increase (*p* = 0.0211), whereas the whey-adapted CM effect did not reach significance. In the lateral hypothalamus (LH), neither of the groups was significantly different from the baseline, but the effect on c-Fos of the Control 20:80 CM was significantly higher than of the 60:40 formulation (*p* = 0.0157). Finally, in the brainstem nucleus of the solitary tract (NTS), a significant above-baseline increase in c-Fos IR was detected after the Control 20:80 CM intake (*p* = 0.005), whereas the higher mean after the 60:40 CM consumption did not reach the threshold of significance. It should be noted, though, that the difference in meal-end NTS activity was detected also between the natural Control 20:80 CM vs. the 60:40 CM formulation (*p* = 0.0396; Figure 3A). Double immunohistochemistry for c-Fos and the anorexigen, OT, revealed that while consumption of either milk type led to an increase in the percentage of activated OT neurons in the SON (20:80 CM *p* = 0.0071; 60:40 CM *p* = 0.0477) and PVN (20:80 CM *p* < 0.001; 60:40 CM *p* = 0.0458), in the PVN consumption of the 20:80 CM produced a significantly higher percentage than the intake of the whey-adapted milk (*p* = 0.0147; Figure 3B). Photomicrographs of sites in which c-Fos levels differed between the 20:80 CM and the whey-adapted formula as well as of the c-Fos-positive OT neurons in the PVN are shown in Figure 4.

**Figure 5:**
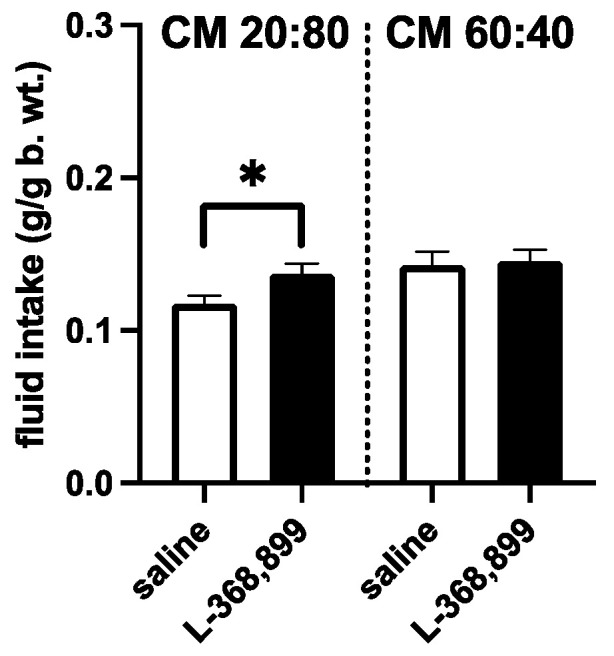
Intake of the individually presented natural Control (20:80 whey:casein) CM and the whey-adapted (60:40 whey:casein) CM in non-deprived mice during 3-h access to the diets. Ten minutes before the meal, the animals were injected with isotonic saline or a blood brain barrier-penetrant OT receptor antagonist, L-368,899 (1 mg/kg). Intake was measured at 3 h. * *p* ≤ 0.05.

Finally, pharmacological blockade of the OT receptor with L-368,899 in nondeprived animals given 3-h access to the Control 20:80 CM significantly increased consumption compared to the saline-injected mice (*p* = 0.0315). On the other hand, the drug treatment had no effect on the intake of the whey-adapted formulation: saline controls and L-368,899-injected animals ingested the same amounts of the 60:40 whey:casein CM (Figure 5).

## 4. Discussion

Establishing a proper level of protein in a diet, including in milk formulations, is driven by the need for adequate delivery of amino acids that facilitate structural integrity and biochemical and signalling processes of the organism. It must balance the avoidance of undesirable effects of excess protein (low acceptability of high-protein foods, hypercalciuria due to protein overload, and in infant nutrition, undesirable effects on kidneys and on weight gain). While one obvious approach is to change the overall dietary protein content, another strategy to improve nutritional and metabolic effects of protein that has been particularly used in cow milk formulations is to adjust the whey:casein ratio. Thus, CM and milk derived from other non-bovine species are oftentimes whey-adapted for human consumption, i.e., the natural whey:casein ratio of 20:80 is adjusted to 60:40. On the one hand, this strategy appears sound as the 60:40 ratio reflects that of human milk. On the other hand, our understanding of the appetitive and metabolic consequences of this switch from the 20:80 to the whey-enhanced content is fragmented: it frequently relies on data that pertain to either whey or casein alone, and not on the 20:80 vs. 60:40 specific ratios [16,19,26,40]. To further complicate matters, CM and other milk (including the second most commonly consumed worldwide, i.e., GM) regardless of being matched for calorie density and macronutrient composition, differ in physical and chemical characteristics.

The current set of studies shows for the first time that whey-adapted CM is consumed more avidly than the natural control 20:80 whey:casein CM. Notably, we observed that a higher intake of the whey-adapted CM occurred regardless of the energy status of the animal, i.e., both calorie-deprived and non-deprived animals given the 60:40 CM as a single-tastant meal ingested more of this milk. The fact that animals readily ingested CM even when they were not hungry indicates that CM at both whey:casein ratios is palatable. It should be noted, however, that in those two feeding scenarios, during the first hour of the meal, consumption of the control 20:80 CM was the same as of the 60:40 CM. It suggests that the motivation to eat was initially similar in both cases. Importantly, it was during the later phase of the meal when differences in ingestive behaviour became apparent. During that time the 20:80 CM controls virtually ceased consumption, but the mice given the whey-adapted CM continued their feeding activity.

While the effect of whey:casein adjustment on single tastant intake bears some similarities between CM and (with what had been previously reported) GM [31], it cannot be directly compared. That is because the earlier report used shorter mealtimes and—in the post-deprivation feeding—utilized simultaneous presentation of GM and chow. It seems though that the enhanced intake of the whey-adapted formulation was achieved in that GM study despite a shorter timeframe of milk presentation (2 rather than 3 h), which may potentially indicate a more robust and immediate effect.

That palatability-driven processes contribute to the intake of the whey-adapted CM is evident through the preference experiments in which animals were given a choice between two kinds of milk. Mice consumed approximately four times more of the 60:40 CM than of the control 20:80 formulation. This four-fold change is a stark difference in preference: as a comparison, earlier studies employing short-term, simultaneous presentations of isocaloric and palatable liquids have shown differences within 10% of the consumed volume [33,34]. Importantly, the 60:40 whey:casein CM formulation was more preferred than the natural 20:80 GM, even though the formulations were similar in energy content and macronutrient composition, and that 60:40 GM had been reported to be more preferred than 20:80 GM in laboratory animals [35]. Our additional preference studies showed that, indeed, when matched for the whey:casein ratios, GM retained its status as the preferred milk. However, enhancing the proportion of whey content superseded the effect of the GM preference reported previously.

A prolonged meal is oftentimes associated with the combination of enhanced palatability of food and delayed satiation. In fact, one of the processes that facilitate enhanced consumption of tasty foods is weakened satiety. For example, at the brain level, it has been shown that agonists of opioid receptors, which mediate eating for palatability by suppressing neural and endocrine mechanisms that promote a feeling of fullness, delay termination of consummatory behaviour, whereas opioid receptor antagonists bring about early satiation, especially when administered in conjunction with the presentation of palatable diets [41,42,43]. This interaction between neural systems mediating reward and satiety occurs primarily at the brain level in the hypothalamus: neural mediators of palatability-driven feeding (e.g., opioids) tend to suppress activation of hypothalamic areas, such as the paraventricular nucleus, which synthesize a number of anorexigenic peptides (including OT) [41,44,45].

Interestingly, our c-Fos expression mapping throughout the brain circuit regulating various aspects of appetite supports the hypothesis of diminished satiety signalling in response to whey-adapted CM contrasted against the natural control 20:80 milk. Compared to the neuronal activation baseline (i.e., at the moment of the presentation of milk), an increase in the number of c-Fos-positive nuclear profiles after CM regardless of the whey:casein ratio was found in the hypothalamic paraventricular nucleus (PVN, the region whose lesion leads to overeating and obesity [46]), which is in line with previous reports examining c-Fos expression after food consumption [47]. Notably, the level of neuronal activation was significantly higher in the 20:80 CM controls than in the whey-adapted group. The dorsomedial hypothalamic c-Fos IR was significantly higher than baseline only in the 20:80 CM- but not in the 60:40 CM-fed mice, whereas in the lateral hypothalamus, the 60:40 CM mice showed lower activation than the natural whey:casein ratio CM-fed controls. A similar pattern of diminished post-meal activity after the 60:40 CM was found in the brainstem nucleus of the solitary tract, which serves as a ‘relay area’ for communication between the periphery (including the gut) and the hypothalamic and extrahypothalamic sites [48,49]. The nucleus of the solitary tract sends projections to, among others, the paraventricular, dorsomedial, and lateral hypothalamic nuclei, thus regions whose activation was affected differently by the natural versus whey-adapted CM [49]. Finally, a similar post-feeding c-Fos response was noted in the ventromedial hypothalamus, a glucose-sensing area [50].

What increases our confidence in stipulating that the satiating effects of the 60:40 CM are subpar compared to the 20:80 formulation is the fact that the percentage of c-Fos positive (activated) OT neurons in the PVN after consumption of the whey-adapted milk is lower than after the intake of the 20:80 CM. OT is known to decrease feeding [32,39]. The strength of an anorexigenic/satiating stimulus is proportional to the level of activation of PVN OT cells [32]. Hence, it is not surprising that prior to the beginning of the meal in this study, the level of OT neuronal activation was very low. It was also expected that regardless of a milk type, after consumption of milk, there would be an increase in OT activity. However, the fact that the ingestion of a similar amount of the 60:40 and 20:80 CM led to a greater increase in the percentage of c-Fos-positive OT neurons after the natural CM suggests that the whey-adapted formula generated a less robust response of the key neuropeptidergic satiety system, thereby facilitating prolonged consumption. This is particularly important considering that the differences in c-Fos expression between the two kinds of milk are seen in the PVN but not in the SON. This is because unlike the SON, which supplies OT to the neurohypophysis, PVN OT neurons send projections not only to the pituitary but also to many brain target areas, including those that regulate reward, gastrointestinal functions, and metabolic processing [36,39].

The involvement of OT in the differential appetitive response to the 20:80 vs. 60:40 CM is further substantiated by the injection study involving the blood-brain barrier penetrant OT receptor antagonist. L-368,899 administration (limiting endogenous OT binding to its receptors) [33,34], increased the intake of the 20:80 CM, but not of the whey-adapted milk.

Among the forebrain sites that mediate primarily eating for pleasure and motivation to eat, both kinds of milk induced activation of the central nucleus of the amygdala, which is in concert with the findings that the control 20:80 and whey-adapted CM are palatable. We did not find differences in the magnitude of the amygdala activation though. Somewhat surprisingly we did not see above-baseline activity in the remaining regions that promote reward (nucleus accumbens core and shell, bed nucleus of the stria terminalis). However, one should note that it may stem from the fact that in order to ensure similar volumes of consumption of both kinds of milk, our paradigm was limited to only 1 h of a meal, which would have likely been extended to 2–3 h should the animals have been allowed to continue feeding. Furthermore, this was an episodic/ad hoc meal rather than a scheduled presentation of palatable food, and the latter would have been more effective in inducing pronounced c-Fos changes in reward areas [51,52].

It should be noted that while whey is often added to products based on ruminant kinds of milk to replicate the 60:40 ratio of human milk, this exacerbates differences in individual proteins, for example, by further increasing the concentrations of beta-lactoglobulin that is not present in human milk (see Table 1). More importantly, the present study demonstrates there are other neuronal consequences of adding whey from ruminant milk. Due to obvious ethical considerations, we were unable to compare these results with human milk. There is evidence of increased weight gain and risk of obesity in infants with a high protein intake associated with formulas with added whey compared to breastfed infants [53]. However, clinical studies have shown that infants fed formula based on GM without whey and at a low protein content have a weight gain [54] and body composition [55] that is comparable to breastfed infants.

In sum, we conclude that the 60:40 whey:casein CM formulation is consumed in greater quantities in prolonged meals by both energy-deprived and non-deprived mice. Mice show a heightened preference for whey-adapted CM in choice scenarios and this phenomenon expands upon GM. Though the intake of the whey-adapted CM induces neural mechanisms promoting satiation, this activation is less pronounced than the one induced by the natural 20:80 whey:casein CM.

## Figures and Tables

**Table 1:** Nutritional composition of CM and GM formulations per 100 mL of prepared solutions. For reference, the last column shows composition of human milk.

Composition (g/100 mL Milk)	Control CM (Natural 20:80 Whey:Casein)	Whey+ CM (60:40 Whey:Casein)	Control GM (Natural 20:80 Whey:Casein)	Whey+ GM (60:40 Whey:Casein)	Human
Energy (kJ)	286.5	273.5	278.1	275.5	275
Protein (g)	1.6	1.4	1.3	1.4	0.97
Lactose (g)	7.3	7.2	7.5	7.1	6.5
Fat (g)	3.8	3.4	3.5	3.5	3.4
αS1 casein (g)	0.43	0.19	-	-	0.039
αS2 casein (g)	0.13	0.06	0.21	0.12	-
B casein (g)	0.54	0.24	0.66	0.38	0.29
κ casein (g)	0.14	0.06	0.1	0.06	0.078
B-lactoglobulin (g)	0.26	0.61	0.22	0.57	-
α-lactalbumin (g)	0.06	0.15	0.08	0.2	0.24
Other whey proteins (g)	0.03	0.08	0.03	0.07	0.31

## Data Availability

The data presented in this study are available on request from the corresponding author.
